# Role of autophagy-related protein in the prognosis of combined hepatocellular carcinoma and cholangiocarcinoma after surgical resection

**DOI:** 10.1186/s12885-021-08553-6

**Published:** 2021-07-17

**Authors:** Daw-Shyong Perng, Chao-Ming Hung, Hung-Yu Lin, Paul Morgan, Yao-Chun Hsu, Tsung-Chin Wu, Pei-Min Hsieh, Jen-Hao Yeh, Pojen Hsiao, Chih-Yuan Lee, Yu-Chan Li, Ya-Chin Wang, Yaw-Sen Chen, Chih-Wen Lin

**Affiliations:** 1 Division of Gastroenterology and Hepatology, E-Da Dachang Hospital, and School of Medicine, College of Medicine, I-Shou University, No. 1, Yida Road, Jiaosu Village, Yanchao District, Kaohsiung City, 82445 Taiwan; 2 Division of Gastroenterology and Hepatology, Department of Medicine, E-Da Hospital, I-Shou University, Kaohsiung, Taiwan; 3 School of Medicine, College of Medicine, I-Shou University, Kaohsiung, Taiwan; 4 Department of Surgery, E-Da Hospital, I-Shou University, Kaohsiung, Taiwan; 5 Department of Surgery, E-Da Cancer Hospital, I-Shou University, Kaohsiung, Taiwan; 6 Department of Surgery, National Taiwan University Hospital, Taipei, Taiwan; 7 School of Chinese Medicine, College of Chinese Medicine, and Research Center for Traditional Chinese Medicine, China Medical University, Taichung, Taiwan

**Keywords:** Combined hepatocellular carcinoma and cholangiocarcinoma, Autophagy, LC3, Prognosis, Predictive factors

## Abstract

**Background:**

Autophagy-related proteins may predict postresection overall survival (OS) and disease-free survival (DFS) in patients with combined hepatocellular carcinoma and cholangiocarcinoma (cHCC-CC).

**Methods:**

We prospectively investigated how these proteins affect clinical prognosis in 40 patients who underwent hepatectomy for cHCC-CC from 2011 to 2019 at a Taiwanese hospital. Levels of autophagy-related proteins, namely LC3, Beclin-1, and p62, were immunohistochemically assessed in patient tumor and non-tumor tissues.

**Results:**

We noted that LC3 expression was significantly correlated with mild clinicopathological characteristics, including macrovascular invasion, lymph node metastasis, American Joint Committee on Cancer and Barcelona Clinic Liver Cancer stages, recurrence, and mortality. Ten patient showed tumor recurrence, and 15 patients died. Postresection 5-year OS and DFS rates were 43.7 and 57.4%, respectively. Cox regression analysis showed that high intratumoral LC3 expression was significantly associated with improved OS [hazard ratio (HR; 95% confidence interval (CI)): (1.68–26.9), *p* = 0.007], but multiple tumors and microvascular invasion was significantly correlated with poor OS [HR (95% CI): 0.03 (0.01–0.34), *p* = 0.004, and 0.07 (0.01–0.46), *p* = 0.006, respectively]. Furthermore, high LC3 expression and cirrhosis had improved DFS [HR (95% CI): 51.3 (2.85–922), *p* = 0.008, and 17.9 (1.05–306), *p* = 0.046, respectively]. The 5-year OS and DFS rates were respectively 61.2 and 74.6% in high LC3 expression patients and 0 and 0% in those with low LC3 expression.

**Conclusion:**

High LC3 expression in tumors is significantly associated with mild clinicopathological characteristics and favorable clinical prognosis in patients with cHCC-CC after resection.

**Supplementary Information:**

The online version contains supplementary material available at 10.1186/s12885-021-08553-6.

## Background

Combined hepatocellular carcinoma and cholangiocarcinoma (cHCC-CC) is a rare type of primary liver cancer that contains unequivocal, intimately mixed components of hepatocellular carcinoma (HCC) and cholangiocarcinoma (CC) [1] . In 1949, Allen and Lisa classified cHCC-CC into three types: types A, B, and C [2]. In type A, HCC and CC are showed at different sites of the liver [3]; in type B, HCC and CC are showed at adjacent sites [3]; and in type C, HCC and CC are combined within the same tumor [3]. This few form of liver cancer is clinically quiescent until the advanced stages, which often manifest with abdominal pain, jaundice, hepatomegaly, and weight loss [3]. cHCC-CC is an extremely aggressive liver cancer that is often associated with poor long-term prognosis [1, 2]. This is largely due to its misdiagnosis as either HCC or CC pre-operatively [4]. The predominant extrahepatic recurrence sites are lymph nodes, which are typically seen in patients with late stage CC [4]. Hence, identification of predictive biomarkers for cHCC-CC overall survival (OS) and disease-free survival (DFS) can help promote the clinical prognosis of patients with cHCC-CC undergoing surgical resection.

The hallmark of autophagy is the formation of autophagosomes, which engulf and break down cytosolic components by fusion with lysosomes [5]. Autophagy-related genes (***ATG***) encode proteins that tightly regulate the process of autophagy [5]. Of the many ATG proteins, ATG8/LC3 is the most studied and has been elucidated as a critical component in autophagosome development. As such, LC3 has been popularized as a marker for monitoring autophagy [6]. LC3 also plays a vital role in cellular differentiation, apoptosis, and cancer development and metastasis [7]. Our previous studies presented that intratumoral LC3 expression and the liver microenvironment are correlated with DFS and OS after surgical resection [7, 8]. However, the expression of LC3 and its possible role in cHCC-CC remain poorly understood and unstudied in the literature.

Beclin-1, another ATG protein, has also been implicated as a biomarker in a variety of tumors. Silencing of the Beclin-1 gene resulted in autophagic dysfunction and ultimately induced spontaneous HCC in mice [9]. Beclin-1 was shown to be poorly expressed in CC, and its expression strongly correlated with lymph node metastasis [10]. Similarly, p62 promoted the selective degradation of deranged proteins by delivering them to autophagosomes [11]. Further, there is overwhelming evidence that p62 is involved in the early stage of cholangiocarcinogenesis [12, 13]. Hence, understanding the expression patterns and alterations in LC3, Beclin-1, and p62 in cHCC-CC can provide new insights into the discovery, diagnosis, and targeting of many autophagy-related human diseases [5]. Finally, the OS and recurrence pattern of cHCC-CC was distinct from that of HCC and CC [14]. Therefore, the aim of this study is to explore the clinicopathological characteristics and risk factors of patients with cHCC-CC and the role of autophagy-related biomarkers for DFS and OS after surgical resection.

## Methods

### Patients and follow-up

Initially, in this prospective cohort study, 608 patients with liver neoplasm who underwent resection from 2011 to 2019 at E-Da Hospital, Taiwan, were recruited. We excluded 568 patients because they had HCC, CC, or metastatic liver tumors. Finally, this prospective study enrolled 40 cHCC-CC patients, which was diagnosed by histology. Our study was approved by the Institutional Review Board of E-Da Hospital (EMRP32100N). Clinicopathological data including demographic features, etiology, cirrhotic liver, tumor behaviors, vascular involvement, metastasis, death, and recurrence were recorded.

Patients were regularly followed up every 3–6 months via abdominal ultrasound, magnetic resonance imaging, or computed tomography. OS was defined as time from the date of cHCC-CC diagnosis to death, the last follow-up, or study completion in June 2019, whichever came first. DFS was defined as time from the date of cHCC-CC diagnosis to recurrence, the last follow-up, or study completion in June 2019, whichever came first.

### Immunohistochemical staining and scoring

Both tumor and non-tumor tissue samples obtained from the patients were formalin-fixed and paraffin-embedded and were confirmed on hematoxylin and eosin-stained sections. The tissues were built as previously described [7, 8, 15, 16]. We stained the tissues with an anti-LC3 antibody (Novus Biologicals, CO, USA), anti-p62 antibody (Abnova, Taipei, Taiwan), and anti-Beclin-1 antibody (Abcam, Cambridge, UK). LC3, p62, and Beclin-1 expression was quantitated by the semiquantitative immunoreactive scoring system (IRS) as previously described [7, 8, 15, 16], and the expression was classified as either negative (IRS < 2) or positive (IRS ≥ 2) according to the percentage and intensity scores (Fig. S1). All the slides were independently calculated by two investigators.

### Data statistics

Categorical data are expressed as numbers and percentages. Continuous data are described as medians and ranges. Student’s *t* test was applied to normally distributed continuous variables, and Wilcoxon rank-sum test was used to compare two groups. The chi-squared test was applied to compare categorical variables. OS and DFS were evaluated by the Kaplan–Meier analysis. Statistically different OS and DFS among groups were done by the log-rank test. Median OS is shown as median and 95% confidence interval (CI). A *p*-value of < 0.05 was regarded statistically significant. All statistical analyses were examined by SPSS version 23.0 (SPSS, Chicago, IL, USA).

## Results

### Demographic features

Overall, 40 cHCC-CC patients were enrolled in our study. The clinicopathological characteristics are shown in Table 1. The median age was 57 years; most patients were male (80%), 40% of patients had hepatitis B virus, 27.5% of patients had hepatitis C virus, and 30% of patients had a history of heavy alcohol consumption. Around 32.5% of patients had cirrhotic liver. Several patients (67.5%) had tumors of ≥5 cm in diameter, and 7.5% of patients had multiple tumors. Approximately 17.5% of patients had macrovascular invasion, and 37.5% of patients had microvascular invasion. Several patients (82.5%) had an American Joint Committee on Cancer (AJCC) stage I/II, and 82.5% of patients had a Barcelona Clinic Liver Cancer (BCLC) stage A/B.

**Table 1:** Demographic characteristics of total patients and relationship between LC3 protein expression

	Total patients, n (%)	LC3 expression		*P*-value
		Low (*n* = 7)	High (*n* = 33)	
Sex				
Male	32 (80.0)	6 (100)	26 (86.7)	0.145
Female	8 (20.0)	1 (0)	7 (13.3)	
Age (years)				
< 60	27 (67.5)	5 (71.4)	22 (66.7)	0.807
≥ 60	13 (32.5)	2 (28.6)	11 (33.3)	
Alcohol use				
Absent	28 (70.0)	4 (57.1)	24 (72.8)	0.062
Present	12 (30.0)	3 (42.9)	9 (27.2)	
HBV positive				
Negative	24 (60)	6 (85.7)	18 (54.5)	0.126
Positive	16 (40.0)	1 (14.3)	15 (45.5)	
HCV positive				
Negative	29 (72.5)	6 (85.7)	23 (69.7)	0.389
Positive	11 (27.5)	1 (14.3)	10 (30.3)	
Cirrhosis				
Absent	27 (67.5)	5 (71.5)	22 (66.7)	0.807
Present	13 (32.5)	2 (28.5)	11 (33.3)	
Edmondson-Steiner Grades				
I-II	29 (72.5)	6 (85.7)	20 (60.6)	0.226
III	11 (27.5)	1 (14.3)	13 (39.4)	
Tumor size				
< 5 cm	13 (32.5)	1 (14.3)	12 (36.4)	0.257
≥ 5 cm	27 (67.5)	6 (85.7)	21 (63.6)	
Tumor number				
single	37 (92.5)	6 (85.7)	31 (93.9)	0.407
multiple	3 (7.5)	1 (14.3)	2 (6.1)	
AFP (ng/ml)				
< 200	29 (72.5)	4 (57.1)	25 (75.8)	0.316
≥ 200	11 (27.5)	3 (42.9)	8 (24.2)	
Resection				
R0	27 (67.5)	5 (71.5)	22 (66.7)	0.807
R1/2	13 (32.5)	2 (28.5)	11 (33.3)	
Microvascular invasion				
Absent	25 (62.5)	3 (42.9)	22 (66.7)	0.237
Present	15 (37.5)	4 (57.1)	11 (33.3)	
Macrovascular invasion				
Absent	33 (82.5)	1 (14.2)	32 (96.9)	< 0.001
Present	7 (17.5)	6 (85.8)	1 (3.1)	
Lympho nodules metastasis				
Absent	33 (82.5)	2 (28.5)	31 (93.9)	< 0.001
Present	7 (17.5)	5 (71.5)	2 (6.1)	
AJCC stage				
I-II	33 (82.5)	2 (28.5)	31 (93.9)	< 0.001
III	7 (17.5)	5 (71.5)	2 (6.1)	
BCLC stage				
A/B	33 (82.5)	3 (42.9)	30 (90.9)	0.006
C	7 (17.5)	4 (57.1)	3 (9.1)	
Recurrence				
Absent	30 (75.0%)	2 (28.6)	28 (84.8)	0.001
Present	10 (25.0%)	5 (71.4)	5 (15.2)	
Mortality				
Absent	25 (62.5)	1 (14.2)	24 (72.8)	< 0.001
Present	15 (37.5)	6 (85.8)	9 (27.2)	

Data are shown as number (percentage). HBV: Hepatitis B virus; HCV: Hepatitis C virus; INR: International normalize ratio; AFP: Alpha-fetoprotein; AJCC: American Joint Committee on Cancer; BCLC: Barcelona clinic liver cancer

### LC3 expression significantly correlated with mild clinicopathological characteristics

Within the cohort, 7 (17.5%) and 33 (82.5%) of the 40 tumor tissues had low and high LC3 expression, respectively, as presented in Table 1. High LC3 expression, compared with low LC3 expression, was remarkably associated with mild clinicopathological characteristics, including macrovascular invasion [6 (85.8%) vs. 1 (3.1%), *p* < 0.001], lymph node metastasis [5 (71.5%) vs. 2 (6.1%), p < 0.001], AJCC stage III [5 (71.5%) vs. 2 (6.1%), p < 0.001], BCLC stage C [4 (57.1%) vs. 3 (9.1%), *p* = 0.006], tumor recurrence [5 (71.4%) vs. 5 (15.2%), *p* = 0.001], and mortality [6 (85.8%) vs. 9 (27.2%), *p* < 0.001].

### LC3 expression is associated with mortality and recurrence

Immunohistochemistry revealed that LC3, Beclin-1, and p62 expression levels were elevated in 82.5% (33 of 40), 62.5% (25 of 40), and 76.5% (27 of 40) of tumor specimens, respectively (Table 2). High intratumoral LC3 expression was remarkably associated with worse survival and low recurrence rate after surgical resection. LC3 expression in non-tumor parts and Beclin-1 and p62 expression in tumor and non-tumor parts was not associated with OS or recurrence.

**Table 2:** Autophagy-related markers in combination hepatocellular carcinoma and cholangiocarcinoma

	Total (*n* = 40)	Non-mortality (*n* = 25)	Mortality (*n* = 15)	*P*-value	Non-recurrence	Recurrence	*P*-value
LC3 in tumors							
Low	7 (17.5)	1 (4.0)	6 (40.0)	0.004	2 (6.7)	5 (50.0)	0.002
High	33 (82.5)	24 (96.0)	9 (60.0)		28 (93.3)	5 (50.0)	
Beclin-1 in tumors							
Low	15 (37.5)	7 (28.0)	8 (53.3)	0.109	9 (30.0)	6 (60.0)	0.09
High	25 (62.5)	18 (72.0)	7 (46.7)		21 (70.0)	4 (40.0)	
p62 in tumors							
Low	13 (32.5)	10 (40.0)	3 (20.0)	0.191	21 (70.0)	8 (80.0)	0.54
High	27 (76.5)	15 (60.0)	12 (80.0)		9 (30.0)	2 (20.0)	
LC3 in ANT							
Low	15 (37.5)	9 (36.0)	6 (40.0)	0.8	10 (33.3)	5 (50.0)	0.346
High	25 (62.5)	16 (64.0)	9 (60.0)		20 (66.7)	5 (50.0)	
Beclin-1 in ANT							
Low	17 (42.5)	10 (40.0)	7 (46.7)	0.68	11 (36.7)	6 (60.0)	0.196
High	23 (57.5)	15 (60.0)	8 (53.3)		19 (63.3)	4 (40.0)	
p62 in ANT							
Low	24 (60.0)	15 (60.0)	9 (60.0)	0.99	20 (66.7)	4 (40.0)	0.136
High	16 (40.0)	10 (40.0)	6 (40.0)		10 (33.3)	6 (60.0)	

ANT: Adjacent non-tumor tissues

### Prognostic factors correlated with OS in cHCC-CC patients underwent surgical resection

**Fig. 1:**
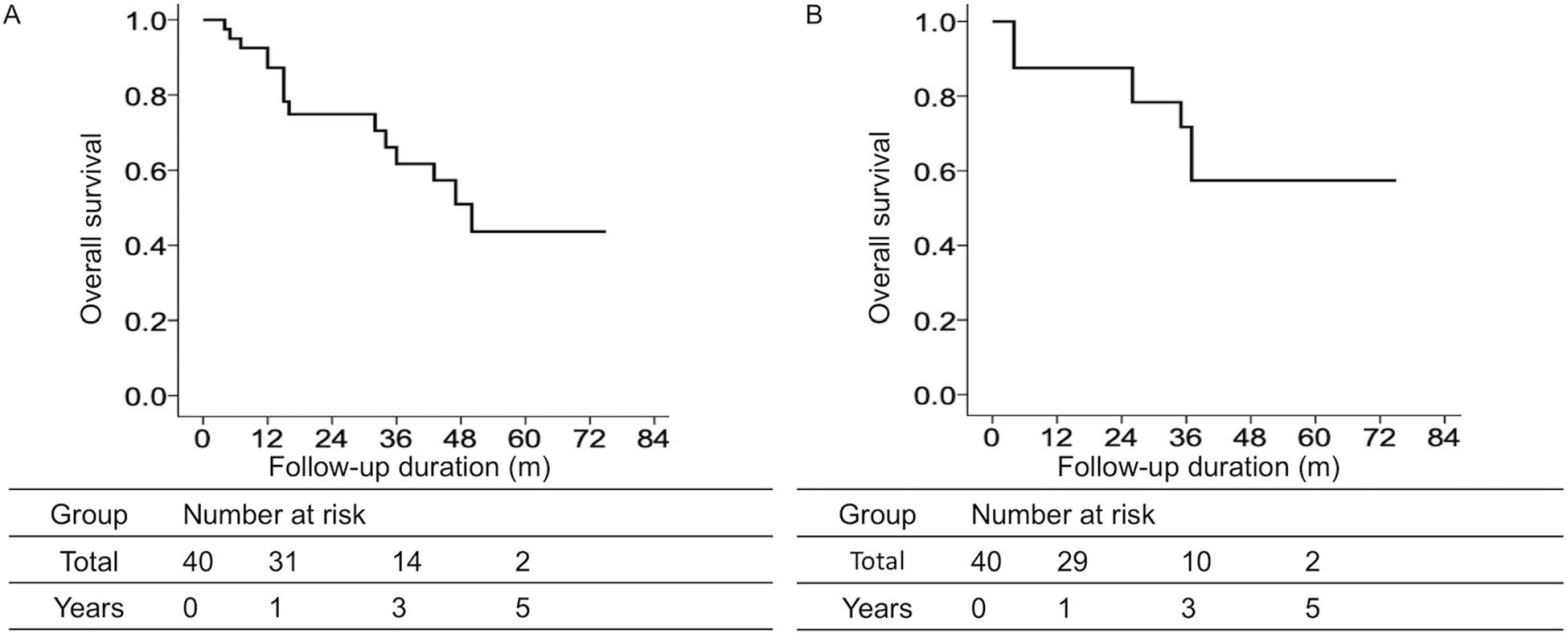
(**A**) Overall and (**B**) disease-free survival in the combined hepatocellular carcinoma and cholangiocarcinoma cohort

The median follow-up duration was 50 months, and 15 cases eventually died. The 1-, 3-, and 5-year OS rates after surgical resection were 87.2, 61.7, and 43.7%, respectively (Fig. 1A). According to the univariate analysis, high intratumoral LC3 expression, microvascular invasion, tumor number, and tumor size were remarkably associated with OS (Table 3).

**Table 3:** Univariate and multivariate Cox regression analyses LC3 for overall survival and disease-free survival of patients in the cohort

**Univariate analysis**	**Overall survival**		**Disease-free survival**	
	Hazard ratio (95% CI)	*P*-value	Hazard ratio (95% CI)	*P*-value
LC3 in tumor (low vs. high)	3.81 (1.35–10.8)	0.012	5.15 (1.47–17.9)	0.01
Sex (male vs. female)	0.47 (0.10–2.20)	0.34	4.85 (1.19–19.7)	0.027
Age (≥60 vs. < 60 years)	2.46 (0.73–8.24)	0.145	2.33 (0.64–8.38)	0.195
Alcohol use (absent vs. present)	0.43 (0.15–1.20)	0.108	3.64 (0.98–13.1)	0.053
HBV (negative vs. positive)	3.16 (0.89–11.8)	0.078	0.76 (0.21–2.73)	0.674
HCV (negative vs. positive)	2.98 (0.37–23.8)	0.303	0.03 (0.01–52.2)	0.36
Cirrhosis (absent vs. present)	1.68 (0.57–4.99)	0.349	0.20 (0.04–1.02)	0.054
Child-Pugh class A				
Edmondson-Steiner Grades (I-II vs. III-IV)	0.88 (0.20–3.95)	0.878	3.93 (0.96–16.0)	0.057
Tumor size (< 5 vs. ≥5 cm)	3.08 (1.19–13.7)	0.037	43.6 (0.18–173)	0.174
Tumor number (single vs. multiple)	0.33 (1.02–1.33)	0.039	0.04 (0.01–12.9)	0.547
AFP (< 200 vs. ≥200 ng/ml)	0.32 (0.08–1.22)	0.095	4.32 (0.94–19.6)	0.059
Resection (R0 vs. R1/2)	1.14 (0.39–3.31)	0.808	3.29 (0.91–11.8)	0.069
Microvascular invasion (absent vs. present)	0.24 (0.08–0.70)	0.009	1.55 (0.42–5.64)	0.506
Macrovascular invasion (absent vs. present)	0.53 (0.11–2.61)	0.44	0.37 (0.00–189)	0.45
Lympho nodules metastasis (absent vs. present)	1.69 (0.46–6.22)	0.425	3.92 (0.96–16.0)	0.057
Distal metastasis (absent vs. present)				
AJCC stage (I-II vs. III-IV)	1.69 (0.46–6.22)	0.425	3.92 (0.96–16.0)	0.057
BCLC stage (A/B vs. C)	0.92 (0.30–2.75)	0.883	2.71 (0.67–10.9)	0.161
Recurrence (absent vs. present)	1.56 (0.47–5.20)	0.466		
**Multivariate analysis**	**Overall survival**		**Disease-free survival**	
	Hazard ratio (95% CI)	*P*-value	Hazard ratio (95% CI)	*P*-value
LC3 in tumor (low vs. high)	6.74 (1.68–26.9)	0.007	51.3 (2.85–922)	0.008
Tumor number (single vs. multiple)	0.03 (0.00–0.34)	0.004		
Microvascular invasion (absent vs. present)	0.07 (0.01–0.46)	0.006		
Tumor size (< 5 vs. ≥5 cm)	3.78 (0.39–36.1)	0.248		
Sex (male vs. female)			15.8 (0.77–322)	0.073
Cirrhosis (absent vs. present)			17.9 (1.05–306)	0.046
Resection (R0 vs. R1/2)			0.77 (0.11–5.05)	0.789

HR: Hazard ratio; CI: Confidence interval; HBV: Hepatitis B virus; HCV: Hepatitis C virus; INR: International normalize ratio; AFP: Alpha-fetoprotein; AJCC: American Joint Committee on Cancer. BCLC: Barcelona clinic liver cancer

The multivariate regression analysis presented that high intratumoral LC3 expression remarkably correlated with improved OS (hazard ratio [HR]: 6.74, 95% Confidence interval [CI]: 1.68–26.9, *p* = 0.007), but multiple tumors and microvascular invasion remarkably correlated with poor OS (HR: 0.03, 95% CI: 0.01–0.34, *p* = 0.004 and HR: 0.07, 95% CI: 0.01–0.46, *p* = 0.006, respectively), as shown in Table 3.

**Fig. 2:**
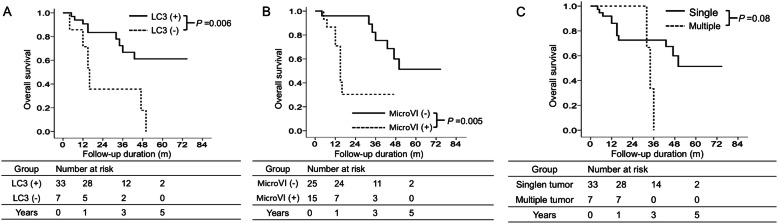
Cumulative incidence of overall survival with respect to various clinicopathological factors using Kaplan–Meier analysis. The cumulative incidence of overall survival is significantly higher in patients with high LC3 expression than in those with low LC3 expression (**A**). The cumulative incidence of overall survival is significantly higher in patients without microvascular invasion than in those with microvascular invasion (**B**). The cumulative incidence of overall survival is not significantly different between patients with single tumor and those with multiple tumors (**C**). MicroVI: Microvascular invasion

Patients with high intratumoral LC3 expression had a remarkably better OS than those with low LC3 expression, as revealed by Kaplan–Meier analysis. The 1-, 3-, and 5-year OS rates were 90.7, 66.8, and 61.2% in high LC3 patients and 71.4, 35.8, and 0% in low LC3 patients, respectively (Fig. 2A). Furthermore, patients with microvascular invasion had a remarkably poorer OS than those without microvascular invasion. The 1-, 3-, and 5-year OS rates were 70.9, 30.4, and 0% in patients with microvascular invasion and 96.0, 75.4, and 51.4% in those without microvascular invasion, respectively (Fig. 2B). However, tumor numbers were not remarkably associated with OS (*p* = 0.08, Fig. 2C).

### Prognostic factors correlated with DFS in cHCC-CC patients underwent surgical resection

Tumor recurrence was observed in 10 patients. The 1-, 3-, and 5-year DFS rates after surgical resection were 87.5, 71.8, and 57.4%, respectively (Fig. 1B). According to the univariate analysis, those factors remarkably correlated with DFS: female, cirrhosis, R0 resection, and high intratumoral LC3 expression.

The multivariate regression analysis revealed that patients with high intratumoral LC3 expression had higher DFS rate (HR: 51.3, 95% CI: 2.85–922, *p* = 0.008) followed by cirrhosis (HR: 17.9, 95% CI: 1.05–306, *p* = 0.046), as presented in Table 3.

**Fig. 3:**
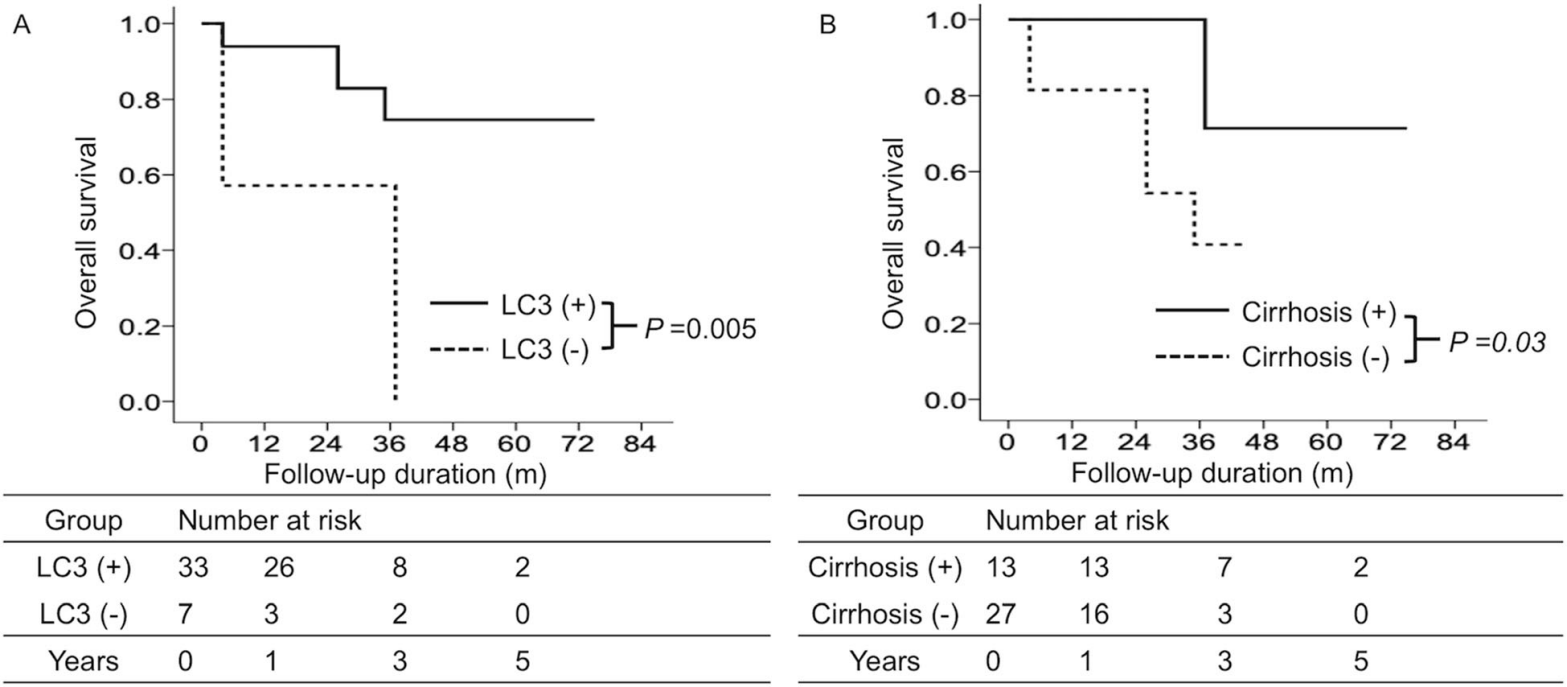
Cumulative incidence of disease-free survival with respect to LC3 expression in tumor and cirrhosis using Kaplan–Meier analysis. The cumulative incidence of disease-free survival is significantly higher in patients with high LC3 expression than in those with low LC3 expression (**A**). The cumulative incidence of disease-free survival is significantly higher in patients with cirrhosis than in those without cirrhosis (**B**)

Patients with high intratumoral LC3 expression had remarkably higher DFS rates than those with low LC3 expression. The 1-, 3-, and 5-year DFS rates were 93.9, 74.6, and 74.6% in high LC3 patients and 57.1, 57.1, and 0% in low LC3 patients, respectively (Fig. 3A). In addition, patients with cirrhosis had remarkably higher DFS rates than those without cirrhosis. The 1-, 3-, and 5-year DFS rates were 100, 100, and 71.4% in patients with cirrhosis and 81.5, 40.7, and 0% in those without cirrhosis, respectively (Fig. 3B).

## Discussion

Although autophagy plays a significant role in HCC and CC development, its role in the clinical outcome of patients with cHCC-CC is not well-understood [7–10, 12, 15]. To the best of our knowledge, this is the first report on the association between autophagy and the clinicopathological significance, prognosis, and clinical outcome of patients with cHCC-CC after surgical resection.

In our study, 40 patients with cHCC-CC who underwent hepatectomy were assessed to identify the predictive factors associated with OS and tumor recurrence. LC3, p62, and Beclin-1 expression levels were elevated in tumors. Moreover, we also noted that high intratumoral LC3 expression was remarkably correlated with mild clinicopathological characteristics and high OS and DFS rates. These results imply that LC3 expression confers protection and serves as a predictive factor of OS and DFS after hepatectomy for cHCC-CC.

Beclin-1 is poorly expressed in CC and is strongly correlated with lymph node metastasis [9, 10]. We previously reported that high intratumoral LC3 expression and the liver microenvironment is associated with mild clinicopathological features in patients with HCC [7, 8]. Here, we found that LC3 expression is significantly correlated with mild clinicopathological features, including macrovascular involvement, lymph node metastasis, AJCC stage, BCLC stage, recurrence, and mortality in cHCC-CC patients. Autophagy is associated with mild clinicopathological features in cHCC-CC patients, similar to that in patients with HCC [7, 8, 15].

Our previous study showed that high intratumoral LC3 expression is remarkably associated with improved OS and DFS after resection in HCC patients. Good clinical outcomes, including OS and DFS, after hepatectomy were found in HCC patients and high LC3 expression [7, 8, 15]. However, Beclin-1 expression in tumors was associated with poor OS and DFS in patients with CC [9, 10]. Our present study demonstrated that high intratumoral LC3 expression is correlated with better OS and DFS rates in cHCC-CC patients. The 5-year OS and DFS rate were 61.2 and 74.6%, respectively, in cHCC-CC patients with high LC3 expression. LC3 expression can predict the clinical outcome of cHCC-CC patients and may have a positive impact on DFS, thus improving OS.

In this study, the multivariate analysis presented that high intratumoral LC3 expression and the presence of multiple tumors and microvascular invasion is remarkably correlated with OS in cHCC-CC patients. Tumor factors (multiple tumors and microvascular involvement) were predictors of poor OS in patients with cHCC-CC. The results are a little different from those in HCC patients, in whom cirrhosis, and tumor recurrence predicted poor OS in our previous study [8].

In the multivariate analysis, high LC3 expression and cirrhosis were found to be correlated with promoted DFS in cHCC-CC patients. Cirrhosis was associated with improved DFS in cHCC-CC patients different from that in HCC patients, who have poor DFS [7]. However, LC3 can be considered an independent predictive factor of DFS and OS in cHCC-CC patients.

This study has some limitations. First, only one-third of our cohort had cirrhosis; this low prevalence may have affected the clinical outcome. Second, the underlying mechanism of autophagy, particularly with respect to the role of LC3 in carcinogenesis and clinical prognosis in cHCC-CC patients, needs to be further explored in vivo and vitro.

## Conclusions

High intratumoral LC3 expression is remarkably correlated with mild clinicopathological characteristics and improved OS and DFS in cHCC-CC patients after surgical resection. Furthermore, this study is the first to show that LC3 expression plays a vital role in predicting OS and DFS in cHCC-CC patients. The analysis of intratumoral LC3 expression, in combination with clinicopathological characteristics, could serve as predictors of OS and DFS after hepatectomy. Our findings indicated that high LC3 expression remarkably associated with mild clinicopathological features and improved OS and DFS in cHCC-CC patients and that LC3 may serve as an important prognostic factor to predict OS and DFS in cHCC-CC patients after surgical resection.

## Supplementary Information

**Additional file 1: Figure S1**. LC3 expression in tumor tissues evaluated by immunohistochemical staining. Representive images according to the proportion of positive cells (**A-D**) and intensity of staining (**E-H**). (**A**) none, (**B**) < 10%, (**C**) 10–50%, (**D**) > 50%; and staining (**E**) absent, (**F**) Weak; (**G**) moderate; (**H**) strong. (upper and lower panel, 400X).

## Data Availability

Data is available from the corresponding author upon reasonable request.

## Abbreviations

cHCC-CC: Combined hepatocellular carcinoma and cholangiocarcinoma
HCC: Hepatocellular carcinoma
CC: Cholangiocarcinoma
AJCC: American Joint Committee On Cancer
BCLC: Barcelona Clinic Liver Cancer
OS: Overall survival
DFS: Disease-free survival
ATG: Autophagy-related genes
IRS: Immunoreactive scoring system
HR: Hazard ratio
CI: Confidence interval
