# Supplementary figures and images for: Role of autophagy-related protein in the prognosis of combined hepatocellular carcinoma and cholangiocarcinoma after surgical resection
Source: BMC Cancer. 2021 Jul 17;21:828. doi: 10.1186/s12885-021-08553-6 (PMC8286562; doi:10.1186/s12885-021-08553-6)

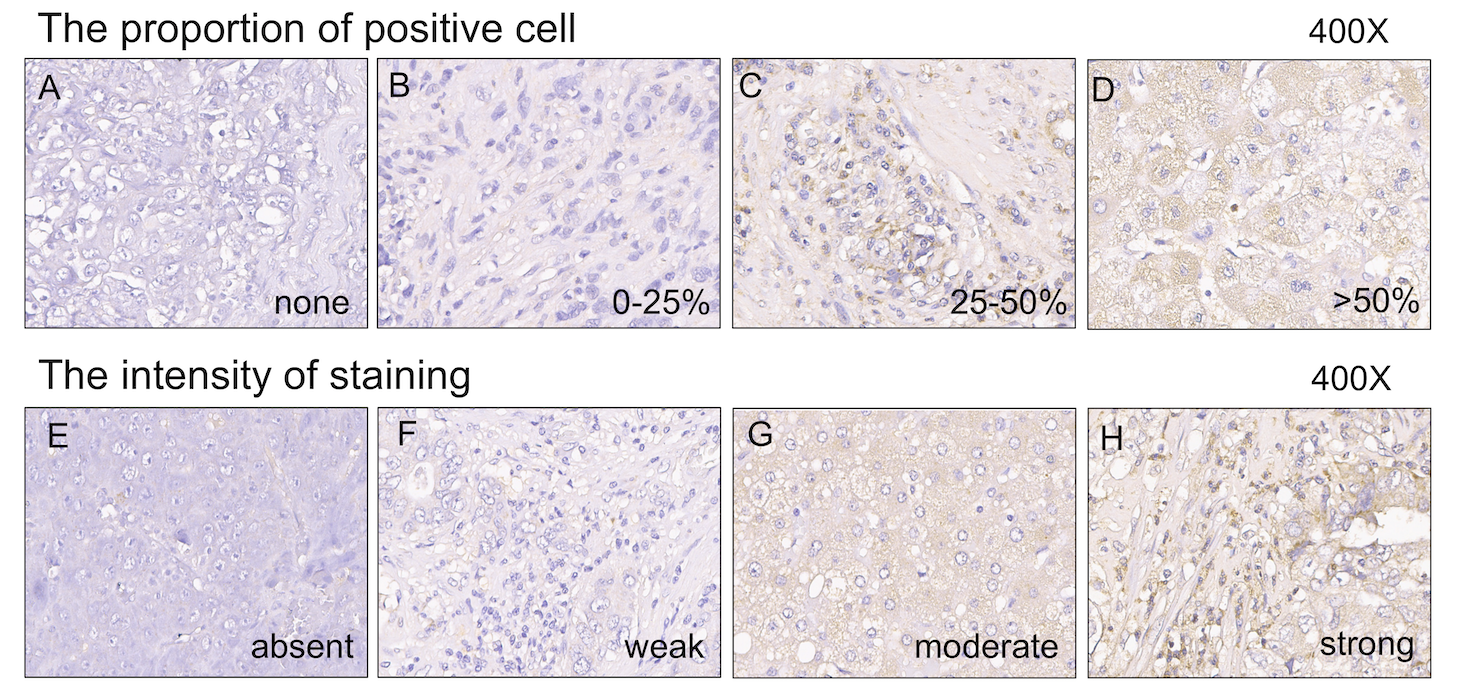

Supplement: Supplementary file 1 — Additional file 1: Figure S1. LC3 expression in tumor tissues evaluated by immunohistochemical staining. Representive images according to the proportion of positive cells (A-D) and intensity of staining (E-H). (A) none, (B) < 10%, (C) 10–50%, (D) > 50%; and staining (E) absent, (F) Weak; (G) moderate; (H) strong. (upper and lower panel, 400X). [file 12885_2021_8553_MOESM1_ESM.png]
